# *Staphylococcus aureus* α-Hemolysin Activates the NLRP3-Inflammasome in Human and Mouse Monocytic Cells

**DOI:** 10.1371/journal.pone.0007446

**Published:** 2009-10-14

**Authors:** Robin R. Craven, Xi Gao, Irving C. Allen, Denis Gris, Juliane Bubeck Wardenburg, Erin McElvania-TeKippe, Jenny P. Ting, Joseph A. Duncan

**Affiliations:** 1 Department of Medicine-Division of Infectious Diseases, University of North Carolina, Chapel Hill, North Carolina, United States of America; 2 Lineberger Comprehensive Cancer Center, University of North Carolina at Chapel Hill, Chapel Hill, North Carolina, United States of America; 3 Department of Microbiology, University of Chicago, Chicago, Illinois, United States of America; 4 Department of Pediatrics, University of Chicago, Chicago, Illinois, United States of America; 5 Department of Microbiology and Immunology, University of North Carolina, Chapel Hill, North Carolina, United States of America; 6 Department of Pharmacology, University of North Carolina, Chapel Hill, North Carolina, United States of America; Columbia University, United States of America

## Abstract

Community Acquired Methicillin Resistant *Staphylococcus aureus* (CA-MRSA) causes severe necrotizing infections of the skin, soft tissues, and lungs. Staphylococcal α-hemolysin is an essential virulence factor in mouse models of CA-MRSA necrotizing pneumonia. *S. aureus* α-hemolysin has long been known to induce inflammatory signaling and cell death in host organisms, however the mechanism underlying these signaling events were not well understood. Using highly purified recombinant α-hemolysin, we now demonstrate that α-hemolysin activates the Nucleotide-binding domain and leucine-rich repeat containing gene family, pyrin domain containing 3 protein (NLRP3)-inflammasome, a host inflammatory signaling complex involved in responses to pathogens and endogenous danger signals. Non-cytolytic mutant α-hemolysin molecules fail to elicit NLRP3-inflammasome signaling, demonstrating that the responses are not due to non-specific activation of this innate immune signaling system by bacterially derived proteins. In monocyte-derived cells from humans and mice, inflammasome assembly in response to α-hemolysin results in activation of the cysteine proteinase, caspase-1. We also show that inflammasome activation by α-hemolysin works in conjunction with signaling by other CA-MRSA-derived Pathogen Associated Molecular Patterns (PAMPs) to induce secretion of pro-inflammatory cytokines IL-1β and IL-18. Additionally, α-hemolysin induces cell death in these cells through an NLRP3-dependent program of cellular necrosis, resulting in the release of endogenous pro-inflammatory molecules, like the chromatin-associated protein, High-mobility group box 1 (HMGB1). These studies link the activity of a major *S. aureus* virulence factor to a specific host signaling pathway. The cellular events linked to inflammasome activity have clear relevance to the disease processes associated with CA-MRSA including tissue necrosis and inflammation.

## Introduction

CA-MRSA is the most commonly identified cause of skin infections seen in emergency rooms in the United States [1]. CA-MRSA can also cause severe, life threatening infections including necrotizing pneumonias and fasciitis, which are associated with very high mortality rates even in previously healthy patients [2], [3]. These necrotizing infections are characterized by localized necrosis and severe inflammation. People colonized with CA-MRSA suffer from some form of *S. aureus* infection at rates about seven times higher than people colonized with other *S. aureus* strains [4]. In addition to resistance to our most commonly used anti-staphylococcal antibiotics, CA-MRSA has increased virulence that is associated with the production of specific factors that assist this pathogen in causing disease [5].

All *S. aureus* produce secreted exotoxin virulence factors including several cytolytic, pore-forming toxins [6]. Both α-hemolysin (Hla) and Panton Valentine leukocidin (PVL) have been implicated in the pathogenesis of *S. aureus* necrotizing pneumonia [7], [8]. Pulmonary delivery of purified α-hemolysin has been shown to induce pulmonary hypertension and inflammation in rat and rabbit [9], [10]. Similar studies in mice with PVL have show that PVL can induce pulmonary inflammation and pneumonitis in mice, in the absence of the bacteria that produce it [8]. More recently, α-hemolysin has been shown to be required for *S. aureus* strains, including CA-MRSA, to promote pneumonia in a mouse model of this disease [7], [11]. Immunization with inactive α-hemolysin protects mice from *S. aureus* pneumonia and prevents production of IL-1β and other cytokines associated with pulmonary exposure to *S. aureus* [12]. Most recently, passive immunization with α-hemolysin-neutralizing antibodies has been shown to protect mice from fatal *S. aureus* pneumonia [12], [13].

In addition to inducing pulmonary inflammation, *S. aureus* α-hemolysin has been shown to induce inflammatory reactions in the eye, skin, and abdomen when injected into rodents [14], [15], [16]. The mechanisms by which α-hemolysin induces inflammation in these intact animals have not been fully evaluated. In endothelial cells, α-hemolysin induces platelet activating factor production [17], [18], [19]. In pulmonary epithelial derived cell lines, exposure to α-hemolysin causes release of nitric oxide and other inflammatory mediators [20]. α-hemolysin has also been shown to act synergistically with *S. aureus* particles to induce some cytokine secretion, including IL-6 and IL-1α in mouse peritoneal macrophages [21]. Finally, α-hemolysin has been shown to induce cell death and IL-1β secretion from human monocytes [22]. The intracellular signaling mechanisms that link α-hemolysin to secretion of cytokines and other inflammatory mediators are unknown.

Physiologic inflammatory signaling can be triggered by pathogen and host derived molecules. The family of nucleotide binding domain and leucine rich repeat containing proteins known as NLR (formerly NALP, NOD, PYPAF, and CATERPILLER) have recently been implicated in several different inflammatory signaling pathways [23], [24]. Mutations in the gene encoding the NLR family member, NLRP3, are associated with hyperactive inflammatory signaling characterized by excessive IL-1β [25], [26], [27]. Many NLR proteins, including NLRP3, have been found to oligomerize into a macromolecular complex known as the inflammasome [28], [29]. The NLRP3-inflammasome is a signaling complex that activates procaspase-1 and induces processing of caspase-1 dependent inflammatory cytokines (particularly IL-1β and IL-18). The NLRP3-inflammasome is activated in response to a multitude of pro-inflammatory stimuli, including many pathogen-derived molecules, endogenous inducers of sterile inflammation, and non-pathogen microbial pore-forming toxins, including nigericin and maitotoxin [30], [31], [32], [33], [34], [35]. Recently, activation of NLRP3 has been found to initiate both caspase-1 dependent cytokine production and a caspase-independent pro-inflammatory program of necrotic cell death [36], [37].

Living *S. aureus* activates NLRP3-mediated signaling in cell culture, but the *S. aureus*-derived factors involved in initiating this signaling remained to be identified [32]. We now show that *S. aureus* α-hemolysin induces NLRP3-mediated signaling triggering caspase-1 activation and programmed necrosis.

## Results

**Figure 1:**
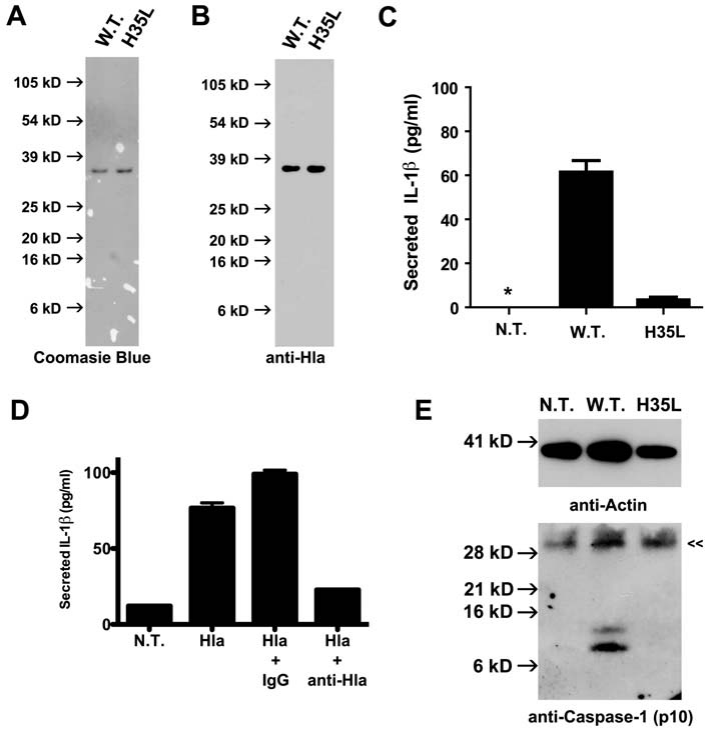
*S. aureus* α-hemolysin induces IL-1β secretion and caspase-1 activation in THP-1 cells. (A) recombinant hexahistidine-tagged α-hemolysin preparations were analyzed by SDS-PAGE and Coomassie Blue staining. Both Wild Type (W.T.) and inactive mutant (H35L) Hla were prepared as described in the materials and methods. (B) The Hla preparations described in (A) were analyzed by immunoblot analysis using polyclonal antisera directed against *S. aureus* α-hemolysin, Abcam product # ab15948. (C) THP-1 cells were untreated (N.T.) or treated with each of the α-hemolysin preparations described in (A) for 1 hour. The cell culture supernatants were analyzed for secreted IL-1β using ELISA. Asterisk (*) denotes cytokine levels below detectable limits (4 pg/ml). (D) Wild type α-hemolysin was prepared with the addition of saline (Hla), control IgG (Hla + IgG), or anti-α-hemolysin IgG (Hla + anti-Hla). THP-1 cells were untreated (N.T.) or treated with the aforementioned α-hemolysin preparations for 1 hour. The cell culture supernatants were analyzed for secreted IL-1β using ELISA. (E) THP-1 cells were either untreated (N.T.) or treated with highly purified recombinant α-hemolysin (W.T. or H35L) for 1 hour, cell lysates were prepared and analyzed by immunoprecipitation and immunoblot analysis for the p10 subunit of activated caspase-1 and actin (loading control) as described in the materials and methods. The double arrow («) indicates the position of protein G used in immunoprecipitation, which binds immunoblot antibodies nonspecifically. Representative data from at least 3 independent experiments are shown.

**Figure 2:**
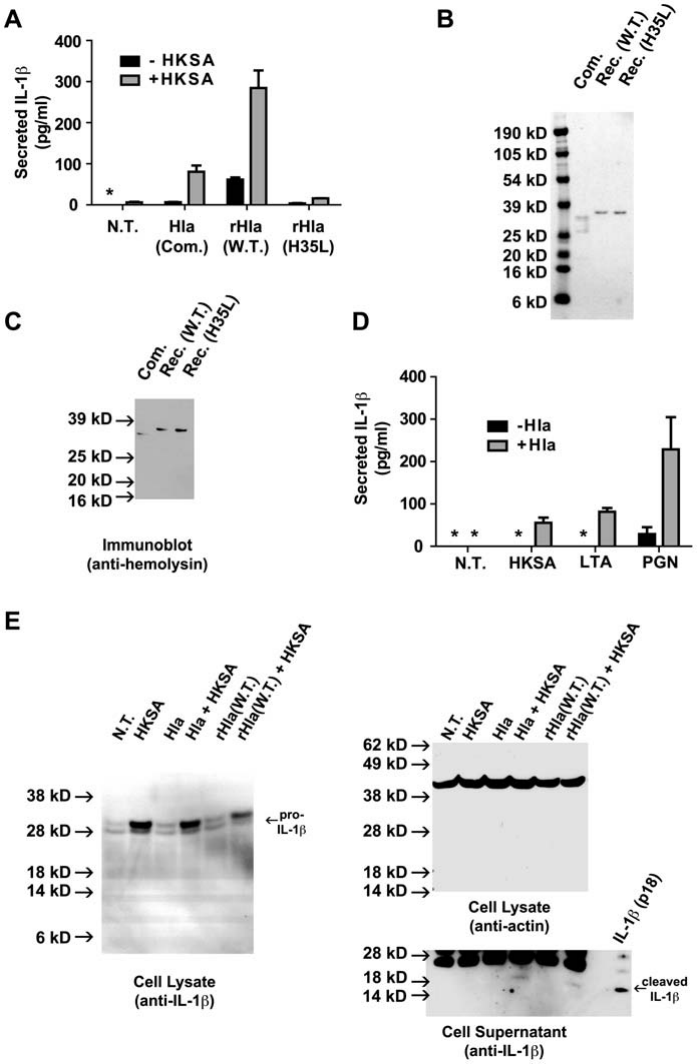
*S. aureus* derived PAMPS cooperate with α-hemolysin in the induction of IL-1β secretion. (A) THP-1 cells were treated with and without Heat Killed *S. aureus* (HKSA, 10^6^ particle/ml) for 3 hours then subsequently treated with 1.0 µg/ml α-hemolysin preparations, commercial (Com.) or recombinant α-hemolysin (rHla) described in Fig. 1, for 1 hour. The cell culture supernatants were subsequently analyzed for secreted IL-1β using ELISA. (B) Commercial (Com.) α-hemolysin from Sigma Aldrich and recombinant (Rec.), hexahistidine-tagged α-hemolysin preparations were analyzed by SDS-PAGE and Coomassie Blue staining. Both Wild Type (W.T.) and inactive mutant (H35L) Hla were prepared as described in the materials and methods. (C) The Hla preparations described in (B) were analyzed by immunoblot analysis using polyclonal antibodies directed against *S. aureus* α-hemolysin (Toxin Technologies product # SLHI101). (D) THP-1 cells were untreated (N.T.) or treated with Heat Killed *S. aureus* (HKSA, 10^6^ particle/ml), *S. aureus* lipotechoic acid (LTA, 100 ng/ml), or *S. aureus* peptidoglycan (PGN, 100 ng/ml) for 3 hours. The cells were subsequently treated either with or without *S. aureus* hemolysin (Hla, 1.0 µg/ml, Sigma Aldrich) for an additional 1 hour. IL-1β secretion into the culture media was measured using ELISA. Asterisk (*) denotes cytokine levels below detectable limits (4 pg/ml). Representative data from at least 3 independent experiments are shown. (E) THP-1 cells were treated as described in (A) with Heat Killed *S. aureus* (HKSA) followed by commercial (Hla) or recombinant α-hemolysin (rHla). Lysates were prepared from the cells and analyzed by immunoblot using antibodies directed against IL-1β (left panel). Lysates were analyzed by immunoblot with antibodies directed against actin (loading control, right/top panel). Processed IL-1β was detected in the cell culture supernatants (right/bottom panel). A mature IL-1β (p18) standard is run in the far right lane.

Prior studies have demonstrated that *S. aureus* α-hemolysin could induce IL-1β secretion in human monocytes [22]. We sought to determine whether we could see similar induction of IL-1β secretion by α-hemolysin in THP-1 cells, a human monocyte-derived cell line. Commercial preparations of α-hemolysin are relatively crude, with manufacturers reporting ∼50% of preparations being protein and the homogeneity of the protein present not reported. In order to study α-hemolysin-induced inflammatory signaling we sought to prepare highly purified *S. aureus* α-hemolysin. Recombinant α-hemolysin bearing a hexa-histidine-tag was prepared after expression in *Escherichia coli*. The recombinant α-hemolysin was isolated from crude *E. coli* homogenates using immobilized metal affinity chromatography. In order to eliminate the possibility of *E. coli* LPS contamination of our preparation, the protein was washed extensively in pyrogen free buffers. After elution, the recombinant protein was exchanged into pyrogen free phosphate buffed saline using size exclusion chromatography. The resulting recombinant α-hemolysin runs as a single homogenous protein band on Coomasie Blue stained SDS PAGE (Fig. 1A). This protein is immunoreactive with multiple anti-α- hemolysin antibodies (Fig. 1B & Fig. 2C). This preparation of α-hemolysin induced IL-1β secretion from THP-1 cells (Fig. 1C). In order to confirm that the ability to activate IL-1β secretion depended on functional α-hemolysin, and not a contaminating substance in the preparation, we prepared an inactive mutant of α-hemolysin (H35L) that lacks hemolytic activity [12]. The mutant α-hemolysin was highly purified, just as the wild-type preparation, yet failed to elicit IL-1β secretion (Fig. 1A, B, & C). A monoclonal antibody that neutralizes α-hemolysin activity and has been shown to protect mice from *S. aureus* pneumonia also reduced IL-1β secretion induced by α-hemolysin when preincubated with the toxin prior to treatment of the cells (Fig. 1D) [13]. In order to demonstrate that α-hemolysin was inducing IL-1β secretion through activation of caspase-1, caspase-1 activation was assessed by immunoblot analysis for the p10 subunit of active caspase-1 after THP-1 cells were treated with wild type or H35L mutant α-hemolysin. Caspase-1 p10 was observed only in cells treated with active α-hemolysin (Fig. 1E). These data all support the hypothesis that α-hemolysin activates IL-1β processing and secretion in a manner similar to other non-pathogen derived pore forming toxins, nigericin and maitotoxin [32].

Unlike preparations of highly purified α-hemolysin, treatment of resting THP-1 cells with commercial preparations of α-hemolysin did not induce detectable levels of IL-1β secretion (Fig. 2A). We sought to further characterize these commercial preparations of α-hemolysin. Using SDS-PAGE and Coomassie Blue protein staining, we found that these preparations contained at least 3 predominant protein bands (Fig. 2B). Immunoblot analysis of these proteins using antibodies directed to α-hemolysin revealed only a single band of immunoreactivity, suggesting the additional proteins within the preparation were derived from other *S. aureus* proteins rather than proteolysis of α-hemolysin (Fig. 2C). These data indicate that significantly less α-hemolysin was added the cells, when compared to highly purified recombinant α-hemolysin.

Production of mature IL-1β is frequently studied in cells pretreated with lipopolysaccharide (LPS) or other Toll-like receptor (TLR) agonists, which are thought to induce expression of pro-IL-1β. Indeed studies demonstrating that secretion of IL-1β could be induced by α-hemolysin were carried out in LPS-treated monocytes [22]. Because *S. aureus* does not produce LPS, we sought to determine whether priming of THP-1 cells with heat killed *S. aureus* (HKSA) could support IL-1β secretion in a manner analogous to LPS treatment. Treatment of THP-1 cells with heat killed *S. aureus* also resulted in minimal IL-1β secretion (Fig. 2A). However, exposure of HKSA-primed THP-1 cells to either commercial or recombinant α-hemolysin induced more robust IL-1β secretion (Fig. 2A). Consistent with its lower α-hemolysin content, the commercial α-hemolysin preparation induced less IL-1β secretion than an equivalent dose of recombinant α-hemolysin. Two *S. aureus* derived TLR ligands [38], [39], [40], peptidoglycan, PGN, (TLR2) and lipotechoic acid, LTA, (TLR4) also caused a synergistic induction of IL-1β secretion in conjunction with α-hemolysin treatment, while causing little or no IL-1β secretion by themselves (Fig. 2D). Immunoblot analysis of cell lysates treated with HKSA, α-hemolysin, or both demonstrated that HKSA did indeed induce production of pro-IL-1β while α hemolysin alone did not (Fig. 2E). Processing of IL-1β, reflected by reduced pro-IL-1β levels and detectable secreted processed IL-1β, could only be detected by immunoblot in the HKSA-primed, α-hemolysin-treated cells (Fig. 2E). These data suggest that α-hemolysin acts downstream of pro-IL-1β synthesis, at the level of processing or secretion, in order to induce IL-1β secretion. Overall, these data demonstrate that multiple *S. aureus*-derived TLR ligands enchance α-hemolysin-induced IL-1β secretion, though they are not required for caspase-1 activation.

**Figure 3:**
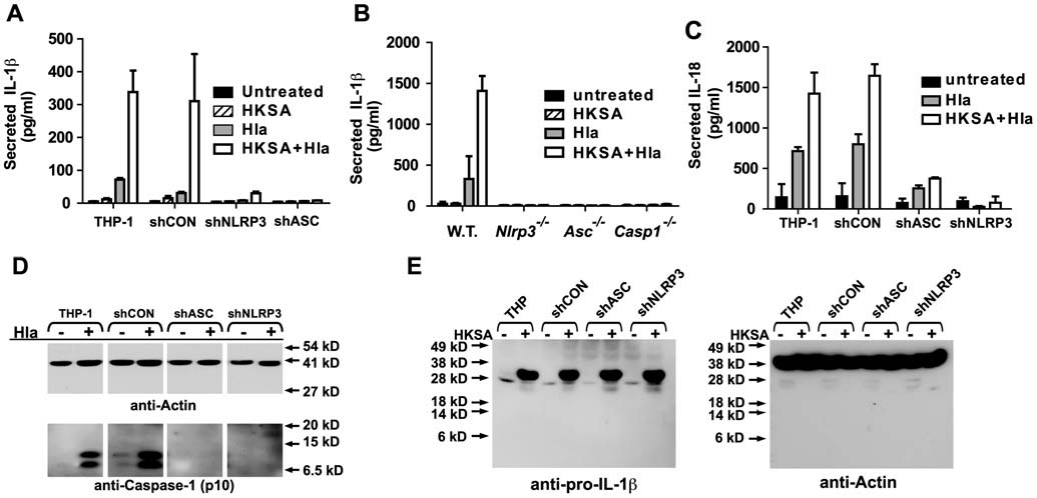
*S. aureus* α-hemolysin induced IL-1β secretion requires the host NLRP3-inflammasome. (A) THP-1-derived cell lines stably transduced with shRNA expressing retrovirus were treated with HKSA (10^6^ particles/ml) or Hla (1.0 µg/ml) for 4 hours or 1 hour, respectively, or a combination of the two, and IL-1β production determined by ELISA. The shRNA's are directed to knock down expression as follows: shCON - negative control (scrambled sequence with base content equal to shASC); shASC – shRNA directed against Apoptotic Speck Containing-protein; shNLRP3 – shRNA directed against NLRP3. (B) Resting peritoneal macrophages were isolated from C57BL/6 mice which were either wild type (WT) or bearing genetic deletion of the NLRP3 (*Nlrp^−/−^*), ASC (*Asc^−/−^*) or Caspase-1 (*Casp1^−/−^*) gene, cultured and treated with HKSA and Hla as described in 2A. Culture supernatant was removed and assayed for the presence of IL1β using ELISA. (C) THP-1-derived cell lines stably transduced with shRNA expressing retrovirus, as described above, were treated with HKSA (10^6^ particles/ml) or Hla (1.0 µg/ml) for 4 hours or 1 hour, respectively, or a combination of the two, and IL-18 secretion determined by ELISA. Representative data from at least 3 independent experiments are shown. (D) THP-1 derived cell lines described in (A) were treated with *S. aureus* α-hemolysin for 1 hour. Cell lysates were analyzed for caspase-1 activation using immunoprecipitation and immunoblot as described in the materials and methods. (E) THP-1-derived cell lines stably transduced with shRNA expressing retrovirus described in (A) were treated with HKSA for 3 hours and cell lysates were analyzed for induction of pro-IL-1β expression using immunoblot analysis (left panel). Equal loading was assessed using immunoblot analysis for actin (right panel). Representative data from at least 3 independent experiments are shown.

The NLRP3 inflammasome has been identified as a critical signaling complex involved in activation of caspase-1 and subsequent IL-1β secretion in response to many inflammatory stimuli, including nigericin and maitotoxin [32], [41]. We sought evidence that the NLRP3 inflammasome was required for α-hemolysin mediated IL-1β secretion. For these studies, we utilized THP-1 derived cell lines that have been transduced with recombinant retrovirus expressing shRNA that “knock down” expression of the inflammasome components, ASC or NLRP3 [36], [42]. When compared to control cell lines expressing no shRNA or a scrambled shRNA, cells lacking expression of either NLRP3 or ASC failed to mount robust IL-1β secretion responses to α-hemolysin treatment (Fig. 3A). To confirm these findings, we carried out a similar set of experiments in peritoneal macrophages from C57Bl/6 mice with genetic knock out of several inflammasome components: *Nlrp3^−/−^*, *Asc^−/−^*, and *Casp1^−/−^*. IL-1β secretion was reduced in HKSA-primed, α-hemolysin-treated peritoneal macrophages derived from *Nlrp3^−/−^*, *Asc^−/−^*, and *Casp1^−/−^* mice when compared to wild-type controls (Fig. 3B). We also sought further evidence that α-hemolysin was inducing IL-1β secretion through NLRP3-inflammasome dependent activation of caspase-1. We tested whether α-hemolysin treatment also led to the secretion of IL-18, another caspase-1 processed cytokine. As expected, control THP-1 cells secreted IL-18 in response to α-hemolysin. Like IL-1β secretion, IL-18 secretion was also enhanced by pretreatment with HKSA. THP-1 cells lacking either NLRP3 or ASC expression failed to secrete significant levels of IL-18 (Fig. 3C). The activation of caspase-1 could be also be detected in THP-1 cells treated with α-hemolysin using immunoblot analysis for the active p10 subunit of caspase-1 in the absence of HKSA-priming. Intact NLRP3 and ASC expression was required for α-hemolysin induced caspase-1 activation as well (Fig. 3D). Immunoblot analysis also confirmed that the THP-1 cell lines lacking NLRP3 or ASC do induce substantial pro-IL-1β production in response to priming with HKSA (Fig. 3E). Together these data suggest that the primary defect in α-hemolysin responsiveness in NLRP3 inflammasome deficient cells is in activation of caspase-1.

**Figure 4:**
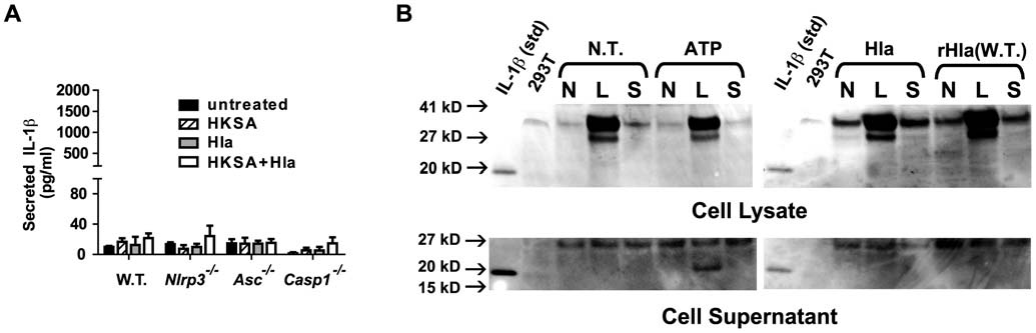
Murine Bone Marrow Derived macrophages are resistant to α hemolysin induced NLRP3-inflammasome activation. (A) Bone marrow-derived macrophages were cultured from the femurs of C57BL/6 mice, which were either wild type (WT), *Nlrp^−/−^*, *Asc^−/−^*, or *Casp1^−/−^*. The macrophages were subsequently pretreated with HKSA or without HKSA for 3 hours then treated for 1 hour with Hla (1 µg/ml) as described in Fig. 2A. Culture supernatant was removed and assayed for the presence of IL-1β using ELISA. Representative data from 3 independent experiments are shown. (B) Bone marrow-derived macrophages were cultured from the femurs of wild type C57BL/6 mice, as described in (A). The cells were then pretreated with nothing (N), LPS (100 ng/ml, L), or HKSA (1×10^6^ particles, H) for 3 hours. The cells were then treated for 1 hour with nothing (N.T.), 1 mM ATP, commercial α-hemolysin (1 µg/ml, Hla), or recombinant α-hemolysin (1 µg/ml, rHla). Cellular lysates and culture supernatants were prepared and subjected to immunoblot analysis with anti-IL-1β antibody.

Mariathasan *et. al.* have demonstrated that bone marrow derived macrophages secrete IL-1β in response to live *S. aureus*. This IL-1β secretion in bone marrow derived macrophages was not significantly reduced when the cells were treated with *S. aureus* lacking α-hemolysin [32]. These reports suggest that either *S. aureus* expresses multiple toxins that activate the NLRP3-inflammasome or that α-hemolysin activates NLRP3 in some but not all monocyte-derived cell lineages. We treated bone marrow derived macrophages with HKSA, α-hemolysin, or the combination of agents and measured IL-1β secretion. Surprisingly, we found wild type, *Nlrp3^−/−^*, and *Asc^−/−^* derived BMDM failed to secrete significant levels of IL-1β to any of these stimuli (Fig. 4A). This suggests that BMDM do not respond to α-hemolysin at the doses used in these studies, unlike the other monocyte-derived cells studied. These BMDM produced pro-IL-1β in response to LPS but not to HKSA preconditioning at the doses used (Fig. 4B). As observed by others, treatment of LPS-primed BMDM with ATP-induced processing of pro-IL-1β and secretion of processed IL-1β [30], [31], [32], [33]. At the same time, α-hemolysin did not activate processing of LPS-induced pro-IL-1β (Fig. 4B). Our data support the fact that α-hemolysin can indeed activate the NLRP3 inflammasome, but suggests that this host response is likely to vary depending on the site of infection and types of host inflammatory cells present to combat it.

**Figure 5:**
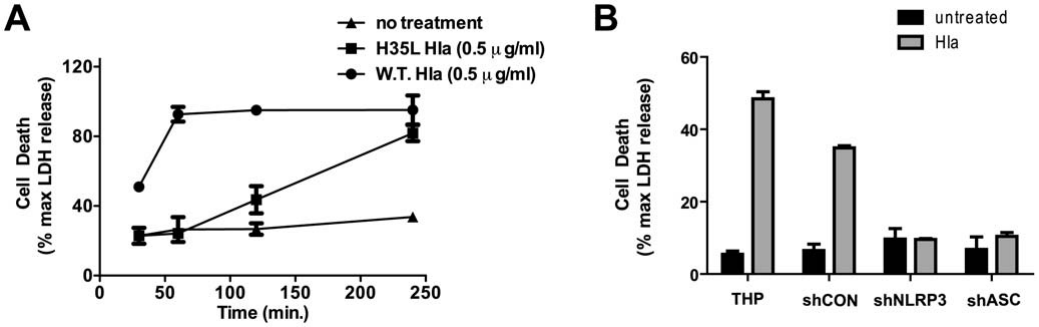
*S. aureus* induces NLRP3-inflammasome- dependent cell death in THP-1 cells. (A) THP-1 cells were treated with 1.0 µg/ml recombinant wild type (W.T.) α-hemolysin or recombinant mutant (H35L) α-hemolysin for 4 hours. Treatment induced cell death was assayed at the indicated time points by measuring LDH activity released into culture supernatant. Levels of LDH are reported as a percent of the maximal LDH activity detected after detergent lysis. (B) THP-1 derived cell lines stably transduced with shRNA expressing retrovirus were treated with or without Hla (1.0 µg/ml, Sigma Aldrich) for 1 hour and cell death was assayed by measuring LDH activity released into culture supernatant. Levels of LDH are reported as a percent of the maximal LDH activity detected after detergent lysis. Representative data from at least 3 independent experiments are shown.

In addition to causing IL-1β secretion, α-hemolysin is known to induce cell death in numerous cell types [22]. We found that α-hemolysin induced death in THP-1 cells in a robust fashion, as demonstrated by release of cytoplasmic LDH into the culture media from THP-1 cells treated with the toxin (Fig. 5A & B.). This activation of cell death was rapid, reaching maximal effect within 60 min. Paralleling our findings in studies of α-hemolysin-induced IL-1β secretion, the mutant α-hemolysin failed to elicit cell death at 1 hour (Fig. 5A), though at longer time points LDH release was observed with the mutant. Also like IL-1β secretion, the induction of cell death in THP-derived cells by α-hemolysin required intact NLRP3 and ASC expression (Fig. 5B).

**Figure 6:**
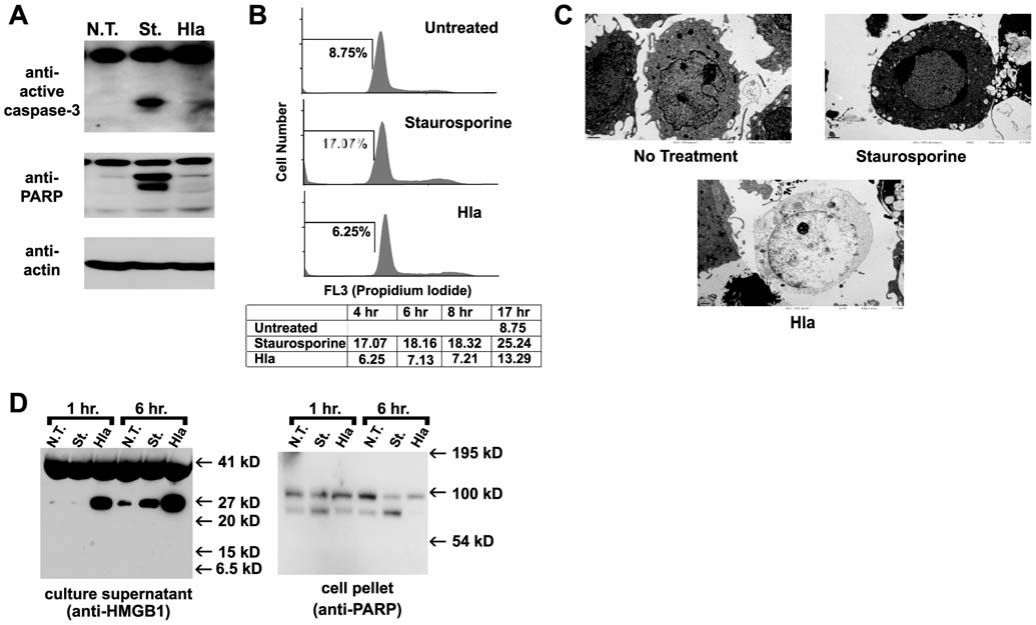
*S. aureus* Hemolysin induces necrotic cell death in THP-1 cells. Lysates from THP-1 cells that were untreated (N.T.) or treated with either Hla (1.0 µg/ml) or staurosporine (1 µM) for 4 hours were subjected to SDS-PAGE and immunoblot with antibodies directed to active caspase-3, Poly ADP ribose polymerase (PARP), or actin. Representative data from 2 independent experiments are shown. (B) THP-1 cells were untreated (N.T.) or treated with either Hla (1.0 µg/ml) or staurosporine (1 µM) over a 17 hour time course. At indicated times the cells were fixed and stained for DNA content with propidium iodide and analyzed using flow cytometry. Cell counts are plotted against propidium iodide fluorescence, which indicates DNA content of the cell. The bars on the histograms indicate the gate for subG1 DNA content (apoptotic cells). Histograms for cells treated for 4 hours are shown, the percent of cells containing subG1 DNA content at 4, 8, 17 hours are charted below the histograms. (C) THP-1 cells, untreated (left) or treated with either Hla (1.0 µg/ml, middle) or staurosporine (1 µM, right), were processed and examined by transmission electron microscopy. Normal cellular morphology is demonstrated in the untreated cells. Morphologic features of necrotic cell death including loss of plasma membrane integrity and cytoplasmic contents as well as intact nuclei with loose chromatin are demonstrated in the Hla treated cells. Apoptotic morphology with nuclear condensation and apoptotic body formation is demonstrated in a staurosporine-treated cell. (D) Culture supernatants from THP-1 cells that were untreated or treated with either Hla (1.0 µg/ml) or staurosporine (1 µM)for 1 and 6 hours were subjected to SDS-PAGE and immunoblot against HMGB1 (left panel). Cell pellets were analyzed for PARP cleavage using immunoblot (right panel). Representative data from at least 3 independent experiments are shown.

*S. aureus* α-hemolysin has been shown to induce both apoptosis and necrotic cell death in peripheral blood mononuclear cells [43], [44]. Our lab and others have recently demonstrated that NLRP3 activation leads to programmed necrosis, termed pyronecrosis [36], [37], [45]. Pyronecrosis is a form of inflammatory cell death and is distinct and independent of the process of apoptosis, which normally results in an orderly disassembly of the dying cells that limits inflammatory responses to the death. In order to confirm that α-hemolysin causes NLRP3-dependent pyronecrosis, we first sought to demonstrate that α-hemolysin-induced death lacked hallmarks characteristic of apoptosis including: activation of caspase-3, cleavage of PARP, and intranucleosomal cleavage of DNA. Immunoblot analysis of lysates from cells treated with α-hemolysin showed no evidence of caspase-3 activation or cleavage of PARP, both of which could be induced in the THP-1 cells by treatment with Staurosporine (which is known to activate apoptosis) (Fig. 6A). Intranucleosomal cleavage of DNA occurs in the setting of apoptosis and is reflected by reduced propidium iodide staining (sub-G1 DNA content) of fixed cells detected by flow cytometry. While staurosporine induced accumulation of sub-G1 DNA content, α- hemolysin treated cells maintained DNA content profiles similar to untreated controls (Fig. 6B). Overall, these studies demonstrate that α-hemolysin-induced NLRP3-mediated cell death lacks traditional apoptotic features.

**Figure 7:**
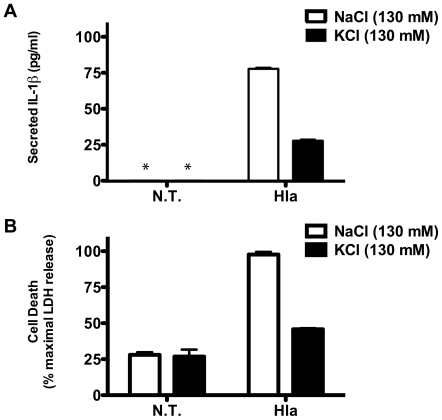
*S. aureus* α-hemolysin requires potassium efflux to induce NLRP3 activation. THP-1 cells were cultured in media containing 130 mM NaCl or 130 mM KCl for 30 min. then treated with and without recombinant α-hemolysin (1 µg/ml) for 1 hour. (A) IL-1β secretion into the culture supernatant was determined using ELISA. (B) Cell death was determined by measuring LDH release and is expressed as a percent of maximum LDH release (determined with detergent lysis). The error bars represent Standard Deviation of triplicate measurements. Asterisk (*) denotes cytokine levels below detectable limits (4 pg/ml). Representative data from 2 independent experiments are shown.

To demonstrate that α-hemolysin induces programmed necrosis in THP-1 cells, we used Transmission Electron Microscopy to examine the morphology of α-hemolysin treated cells. The α-hemolysin treated cells lacked morphologic features of apoptotic cells seen in staurosporine treated cells, including chromatin condensation and blebbing of apoptotic bodies from the plasma membrane (Fig. 6C). Instead they have necrotic morphology with loss of plasma membrane integrity and cytoplasmic contents as well as intact nuclei with loose chromatin. Necrotic cell death results in the release of the chromosomal protein HMGB1. Release of HMGB1 is also observed from apoptotic cells in tissue culture as a result of secondary necrosis, but this occurs at a much later time point than is seen in primary necrosis [36]. Immunoblot analysis of cell culture supernatants from α-hemolysin treated THP-1 cells demonstrates that α-hemolysin induced death is accompanied by early release of HMGB1 protein, which is not seen in staurosporine treated cells (Fig. 6D). Staurosporine-induced apoptotic death was confirmed in these cultures by immunoblot analysis for PARP cleavage (Fig. 6D). Prior studies have demonstrated that induction of IL-1β processing through activation of the NLRP3-inflammasome in response to the pore-forming toxin Nigericin requires intracellular potassium depletion. Both NLRP3-inflammasome dependent IL-1β secretion and necrotic cell death were inhibited when cells were treated with *S. aureus* α-hemolysin in the presence of 130 mM KCl (Fig. 7A & B). This data, combined with the inability of mutant α-hemolysin to induce IL-1β secretion or rapid necrosis, suggests that *S. aureus* α-hemolysin activates the inflammasome through potassium depletion and not through direct interaction with other host signaling proteins. In total, this work demonstrates that α-hemolysin actually requires host NLRP3 signaling in order to induce necrotic cell death.

**Figure 8:**
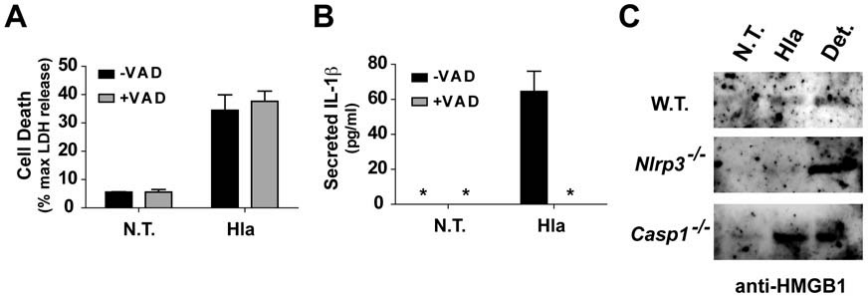
*S. aureus* α-hemolysin induced necrotic cell death is independent of caspase-1. (A & B) THP-1 cells were pretreated with the pan-caspase inhibitor Z-VAD(OMe)-FMK (+VAD) or DMSO vehicle (-VAD). The cells were then divided and either treated or not treated with α-hemolysin. Cell culture supernatants were analyzed for cell death using LDH activity (A) and secreted IL-1β using ELISA (B). Asterisk (*) denotes cytokine levels below detectable limits (4 pg/ml). Representative data from at least 3 independent experiments are shown. Cell death is reported as percent of maximal lysis observed with detergent above LDH levels present in sterile culture media. (C) Resting peritoneal macrophages were isolated from C57BL/6 mice, either wild type (WT) or bearing genetic deletion of the NLRP3 (*Nlrp^−/−^*) or Caspase-1 (*Casp1^−/−^*) gene, and left untreated (N.T.) or treated with recombinant Hla (0.5 µg/ml) for 4 hours. Culture supernatant was removed and analyzed for the presence of HMGB1 using immunoblot analysis (as described in Fig. 6). Detergent (Det.) lysis was carried out as an internal positive control for HMGB1 release in cells from each genotype for each experiment. Representative immunoblots are presented from two independent experiments.

Though the NLRP3 inflammasome activates the cysteine proteinase, caspase-1, NLRP3- dependent programmed necrosis has been shown to be independent of caspase activation. Pretreatment of THP-1 cells with a cell permeable pancaspase inhibitor did not block α-hemolysin induced cell death (Fig. 8A). We confirmed the inhibitor was functioning by examining α-hemolysin-mediated IL-1β secretion in the treated cells, which was completely abrogated by treatment with caspase inhibitor (Fig. 8B). We also studied induction of pyronecrosis in peritoneal derived macrophages treated with α hemolysin. While α-hemolysin induced release of HMGB1 into the culture supernatant at levels equivalent to detergent induced lysis from wild type peritoneal macrophages, *Nlrp3^−/−^* macrophages were resistant to α-hemolysin induced HMGB1 release (Fig. 8C). Consistent with our findings using caspase inhibitors, release of HMGB1 was induced by α-hemolysin in *Casp1^−/−^* macrophages (Fig. 8C). Combined, these data confirm that α-hemolysin induced pyronecrosis is independent of caspase-1 activation and IL-1β secretion, which is consistent with reports from other groups.

## Discussion

We have demonstrated that highly purified recombinant *S. aureus* α-hemolysin is capable of inducing activation of caspase-1 through the NLRP3 inflammasome. Activation of NLRP3 by α-hemolysin induces cytokine processing and programmed necrosis, both of which can explain many of the pro-inflammatory properties of α-hemolysin that have been observed in the past. In fact, these studies identify the mechanism by which α-hemolysin induces IL-1β secretion, an observation that was published almost twenty years ago [22].

In the initial report of *S. aureus* activating NLRP3-mediated IL-1β secretion, Mariathasan *et. al*. found that *S. aureus* lacking α-hemolysin, β-hemolysin, or γ- hemolysin still activated IL-1β secretion [32]. Our findings using purified toxin clearly demonstrate that NLRP3 is activated by α-hemolysin. Because bone marrow derived macrophages do not respond robustly to α-hemolysin in our studies, the findings of Mariathasan *et al*. are not surprising. Additionally, given the large number of pore-forming and membrane disrupting toxins produced by *S. aureus*, it seems likely *S. aureus* may have redundant factors that activate NLRP3-signaling. Supporting this possibility, we have found that other pore-forming *S. aureus* toxins (particularly Panton Valentine Leukocidin) also induce NLRP3-dependent cytolysis (data not shown). Since some of these toxins exert differential cytolytic activity based on cell type, the ability to activate NLRP3-mediated signaling may be important in establishing infection in a variety of infection sites [22].

The ability of α-hemolysin to activate programmed cell death in host cells has been the subject of numerous studies. In early studies, α-hemolysin was shown to activate apoptotic machinery in studies carried out primarily in lympocyte derived cell lines [46]. This report demonstrated that apoptotic caspases were activated and down stream apoptotic effects were observed, including cleavage of α-fodrin and intranucleosomal DNA cleavage. However, these effects were studied at times between 4 and 24 hours. A second report by this group later demonstrated that α-hemolysin induced cell death, as measured by loss of membrane integrity, occurred even if apoptotic machinery was inhibited [44]. Additionally, they demonstrated that α-hemolysin induced death was accompanied by morphologic features of necrosis and release of HMGB1 into the culture supernatant. We now show that pyronecrosis is activated very rapidly by α-hemolysin, in a timeframe earlier than has previously been characterized for α hemolyin-induced death and with features consistent with these other reports. Namely, we report α-hemolysin induced death is independent of caspase-activation, has morphologic features of necrosis, and is accompanied by early release of HMGB1. Interestingly, when taken out to 16 hours, we did observe some cells with sub-G1 DNA content, which may represent activation of apoptotic machinery in cells from the population that resisted activation of pyronecrosis. Overall, we have demonstrated that, in monocytic cells, activation NLRP3-dependent pyronecrosis by α-hemolysin is the primary mechanism underlying the cytolytic activity of the toxin.

The activation of host NLRP3 by α-hemolysin requires the hemolytic activity of the toxin. Our data demonstrates that mutations in the toxin that eliminate hemolytic activity also eliminate the toxin's cytolytic activity in monocyte derived cells and ability to induce NLRP3-inflammasome signaling. A recent study of a similar α-hemolysin mutant demonstrates that this mutant toxin is capable of inducing an apoptotic host response [47]. Our experiments also demonstrate that although mutant α-hemolysin does not induce NLRP3-dependent IL-1β secretion, it does induce cell death with a time delay (Fig. 5). We suspect that this death may represent apoptotic death observed by others using mutant α-hemolysin, though formal demonstration of this requires further investigation. Interestingly, this mutation also renders the toxin incapable of causing death in animal models of α-hemolysin induced peritonitis, presumably due to reduced activation of inflammatory signaling [48]. The α-hemolysin gene, *Hla*, is a critical virulence factor in cellulitis, mastitits, and pneumonia models of *S. aureus* infection [49], [50]. We speculate that activation of inflammatory signaling through the inflammasome may be essential for α -hemolysin to mediate virulence. In support of this hypothesis, α-hemolysin deficient *S. aureus* fail to establish lethal infection or induce significant pulmonary infection [7], [11]. Additionally, immunization with mutant α-hemolysin protects mice from *S. aureus* pneumonia and reduces IL-1β production in the lungs of the challenged mice [12]. Furthermore, we have now shown a monoclonal antibody, which is protective against *S. aureus* pneumonia in passive immunization studies of mice, inhibits NLRP3-mediated signaling in cultured cells (Fig. 1D) [13]. It is certainly possible that host inflammatory responses and programmed necrosis could damage host tissue architecture and compromise the innate capacity of these tissues to resist infection. Further experimentation is clearly required to determine the role of NLRP3 inflammasome signaling in the pathogenesis of *S. aureus* infection. If this hypothesis is born out, inhibition of inflammasome signaling may turn out to be a useful adjuvant therapy to appropriate antibiotic use in severe *S. aureus* infections.

## Materials and Methods

### Ethics Statement

All protocols used in this study were approved by the Institutional Animal Care and Use Committees at the University of North Carolina.

### Preparation of recombinant α-hemolysin

*E. coli* (BL21/DE3) harboring a hexahistidine-tagged *S. aureus* α-hemolysin (wild type and H35L mutant)-expressing plasmid, construction described by Ragle and Bubeck Wardenburg [13], were grown overnight in LB supplemented with kanamycin (50 ug/ml). Overnight cultures were diluted 1∶10 into 100 ml LB with kanamycin (50 ug/ml) and grown for 3 hours. α-hemolysin expression was induced by addition of IPTG to 1 mM. After 2 hours at 37°, the *E. coli* were harvested by centrifugation and lysed in Dulbecco's Phosphate Buffered Saline (D-PBS) supplemented with complete protease™ inhibitor cocktail (Roche, product # 04 693 116 001) by sonication. The lysate was clarified by centrifugation at 13,000 x g for 30 min and applied to immobilized nickel resin (PrepEase Histidine-tagged Protein Purification Sample Kit, USB, product # 78810). The bound protein was washed extensively (50 column volumes) with pyrogen free tissue culture grade D-PBS (Invitrogen/Gibco, product # 14040-133) and eluted in D-PBS supplemented with 200 mM imidazole. Imidazole was removed from the eluted proteins by size exclusion chromatography: 2 sequential passages through a sephadex G-25 (Disposable PD-10 Desalting Columns, G.E. Healthcare, product # 17-0851-01) equilibrated with pyrogen free PBS. Final protein preparations concentration was determined using Bradford protein assay reagent (Bio-Rad Protein Assay, Bio-Rad, product # 500-0006) with albumin protein standards, purity of the proteins was assessed using SDS-PAGE analysis.

### Mouse strain maintainance and care

The generation of *Nlrp3^−/−^*, *Asc^−/−^*, and *Casp1^−/−^* mice has been described previously [30], [51]. The mice were kindly provided by Millenium Pharmaceuticals and Dr. Vishva Dixit at Genentech, and Dr. Richard Flavell at Yale University, respectively. The mice have been backcrossed onto the C57BL/6J genetic background for at least nine generations. Age- and sex-matched C57BL/6J mice purchased from The Jackson Laboratory (Bar Harbor, ME) were used as wild-type controls. The mice were maintained according to institutional policies.

### Culture of mammalian cells

The construction of THP-1-derived cell lines expressing shRNA targeting NLRP3 and ASC as well as control cell lines using recombinant retrovirus has been previously described [36], [42]. All cell lines were maintained in RPMI Media 1640 (Invitrogen/Gibco, product # 12633-012) supplemented with 10% fetal calf serum and penicillin and streptomycin. Resting peritoneal macrophages were obtained by peritoneal lavage of euthanized mice with Hanks Buffered Saline supplemented with 10% fetal calf serum and penicillin/streptomycin. Lavaged cells were counted and plated at a density of 2×10^6^/ml in 96 well tissue culture plates. Bone marrow-derived macrophages were prepared as previously described [36]; briefly, bone marrow was harvested by necroptic dissection of euthanized mice. The cells were resuspended in 25 mls/femur DMEM supplemented with 10% FBS (Hyclone Characterized Fetal Bovine Serum, Thermo Scientific, product # SH30071), L-glutamine (Invitrogen/GIBCO, product # 21051-024), 100 µM Non-Essential Amino Acids (Invitrogen/GIBCO, product # 11140-050), 20% L929 conditioned media, and plated at 12.5 ml/100×20 mm non-tissue culture treated plates. The adherent bone marrow-derived macrophages were cultured in DMEM with 20% L929 cell supernatant (as a source of M-CSF) for 7 days. The cells were then treated as indicated (treatments detailed below.)

### Treatment of cultured cells with α-hemolysin

When indicated cells were preincubated with Heat Killed *S. aureus* (HKSA, Invivogen, product # tlrl-hksa) at 1 particle/cell (typically 10^6^/ml) for 3 hours. *S. aureus* α-hemolysin (Sigma-Aldrich, product # H9395) or recombinant α-hemolysin (preparation described above) was added to the cells for 1 hour. Soluble culture supernatants and cell pellets were collected after centrifugation at 13,000 xg for 10 min. LDH activity was measured in supernatants using Cyto-tox ONE kit (Promega, product # G7891) and a Fluorostar fluorescence plate reader (BMG Technologies). IL-1β and IL-18 were measured in culture supernatants using ELISA kits (R & D Systems, product # DLB50 and 7620) according to the manufacturer's protocols. When staurosporine treatment was used as a positive control for apoptotic cell death, staurosporine solution (1 mM) was obtained from Sigma-Aldrich (product # S6942) and added to the culture to final concentration of 1 µM. When caspase inhibitors were used, Z-VAD(OMe)-FMK, (Alexis Biochemicals, product # ALX-260-039) was added to the cells to a final concentration of 20 µM from a stock of inhibitor in DMSO at a concentration of 20 mM. A similar volume of DMSO (1 µg/1 ml) was added to the control cells. Cell pellets were fixed in 2% paraformaldehyde, 2.5% gluteraldehyde, 0.15 M sodium phosphate, pH 7.4 solution for studies involving electron microscopy.

### Immunoblot analysis

Cell pellets were lysed using PBS supplemented with 1% Igepal, protease inhibitor, 2 mM DTT and soluble lysates for immunoblot analysis were generated by centrifugation at 13,000 xg for 15 min. When indicated, culture supernatants are run on SDS PAGE for analysis. Invitrogen precast gels (NuPAGE) were used for all gel-based analyses. Proteins were transferred to nitrocellulose using the Invitrogen iBlot system and immunoblot analysis carried out using antibodies directed against: human and mouse IL-1β (R &D Systems, product # AF-201-NA & AF-401-NA, respectively), anti – active caspase-3 (Cell Signaling, product # 9662), anti-PARP (Cell Signaling, product # 9542), anti-HMGB1 (Abcam, product # ab18256), actin (Santa Cruz Biotechnology, product # sc-8432 HRP). HRP-conjugated anti-mouse and anti-rabbit secondary antibodies were from Santa Cruz Biotechnology.

Activation of caspase-1 was assessed using an immunoprecipitation/immunoblot analysis previously described [52]. Cells were cultured at 10^7^ cells/ml and treated as indicated. Cellular lysates for analysis of caspase-1 p10 were prepared by the addition of proteinase inhibitors (complete™, Roche, product # 04 693 116 001) and NP-40 (final concentration 0.1%) to the treated cell cultures followed by centrifugation at 13,000 xg for 10 min. Caspase-1 and actin were then simultaneously immunoprecipitated by incubation with antibodies to caspase-1 p10 (Santa Cruz Biotechnology, sc-515) and actin (Santa Cruz Biotechnology, sc-7210) from Santa Cruz Biotechnology and Ultralink protein G (Thermo Scientific/Pierce, product # 53125) overnight at 4°C. The immunoprecipitated proteins were analyzed by immunoblot with antibodies directed to caspase-1 (Imgenex, product # IMG-5028) and actin (Santa Cruz Biotechnology, sc-8432 HRP).
